# A Split-Ubiquitin Two-Hybrid Screen for Proteins Physically Interacting with the Yeast Amino Acid Transceptor Gap1 and Ammonium Transceptor Mep2

**DOI:** 10.1371/journal.pone.0024275

**Published:** 2011-09-02

**Authors:** Griet Van Zeebroeck, Marlies Kimpe, Patrick Vandormael, Johan M. Thevelein

**Affiliations:** 1 Laboratory of Molecular Cell Biology, Institute of Botany and Microbiology, KULeuven, Flanders, Belgium; 2 Department of Molecular Microbiology, The Vlaams Instituut voor Biotechnologie, Flanders, Belgium; Institute of Developmental Biology and Cancer Research, France

## Abstract

Several nutrient permeases have been identified in yeast, which combine a transport and receptor function, and are called transceptors. The Gap1 general amino acid permease and the Mep2 ammonium permease mediate rapid activation by amino acids and by ammonium, respectively, of the protein kinase A (PKA) pathway in nitrogen-starved cells. Their mode of action is not well understood. Both proteins are subject to complex controls governing their intracellular trafficking. Using a split-ubiquitin yeast two-hybrid screen with Gap1 or Mep2 as bait, we identified proteins putatively interacting with Gap1 and/or Mep2. They are involved in glycosylation, the secretory pathway, sphingolipid biosynthesis, cell wall biosynthesis and other processes. For several candidate interactors, determination of transport and signaling capacity, as well as localization of Gap1 or Mep2 in the corresponding deletion strains, confirmed a functional interaction with Gap1 and/or Mep2. Also common interacting proteins were identified. Transport and signaling were differentially affected in specific deletion strains, clearly separating the two functions of the transceptors and confirming that signaling does not require transport. We identified two new proteins, Bsc6 and Yir014w, that affect trafficking or downregulation of Gap1. Deletion of *EGD2*, *YNL024c* or *SPC2* inactivates Gap1 transport and signaling, while its plasma membrane level appears normal.. Vma4 is required for Mep2 expression, while Gup1 appears to be required for proper distribution of Mep2 over the plasma membrane. Some of the interactions were confirmed by GST pull-down assay, using the C-terminal tail of Gap1 or Mep2 expressed in *E.coli*. Our results reveal the effectiveness of split-ubiquitin two-hybrid screening for identification of proteins functionally interacting with membrane proteins. They provide several candidate proteins involved in the transport and signaling function or in the complex trafficking control of the Gap1 and Mep2 transceptors.

## Introduction

In the yeast *Saccharomyces cerevisiae*, several nutrient transporters have been identified which combine their transport function with an additional signaling function for rapid activation of the protein kinase A (PKA) pathway in cells starved for the nutrient substrate of the transporter. Prominent examples are the amino acid permease, Gap1, and the ammonium permease, Mep2, which mediate amino acid and ammonium activation, respectively, in nitrogen-starved cells [1], [2]. The phosphate carriers Pho84, and to a minor extent Pho87, play a similar role for phosphate activation of the PKA pathway in phosphate-starved cells [3]. Recent work has identified non-transported agonists of the signaling function of Gap1 and Pho84 and it was shown that the same substrate-binding site is used both for transport and signaling [4], [5]. The transceptors seem to use a non-classical pathway for activation of PKA since cAMP is not required as second messenger in nitrogen and phosphate activation [3], [6], [7].

Although less well established, evidence was obtained for a transceptor function of Mep2 and Mep1 in mediating ammonium activation of the PKA pathway [1], [8]. Mep2 is also required for induction of pseudohyphal growth [9]. Pseudohyphal growth is induced under nitrogen limitation and requires two pathways: the mitogen activated protein kinase (MAPK) pathway and the cAMP-PKA pathway (reviewed by [10]). The precise connection between Mep2 and these two pathways remains unclear, although recent results confirm that Mep2 functions as a transceptor triggering pseudohyphal growth upstream of the MAPK pathway rather than the PKA pathway [11]. The Sch9 protein kinase is required for nitrogen but not for phosphate activation of the pathway [3]. How it functions precisely in the signaling pathway is not yet clear. Genome-wide expression analysis revealed that Sch9 most probably acts in a parallel, partially redundant pathway with PKA [12].

The Gap1 amino acid permease has been studied in great detail as a model system for regulation of nutrient transporter trafficking. Gap1 is a very promiscuous permease that transports most biological amino acids, as well as many D-amino acids and amino acid analogs. The synthesis of both Gap1 and Mep2 is tightly regulated by the nitrogen catabolite repression (NCR) pathway according to the quality of the nitrogen source present in the medium [13]. *GAP1* and *MEP2* expression is strongly induced during nitrogen starvation and during growth on poor nitrogen sources by the transcriptional activators Gln3 and Gat1/Nil1 [14], [15]. Under these conditions, Gap1 and Mep2 accumulate at the plasma membrane in an active, stable form. In some way, Npr1 kinase activity positively regulates their activity by post-translational modification [16], [17]. Addition of amino acids triggers ubiquitination, endocytic internalization and breakdown of Gap1 in the vacuole-lysosome of the yeast cells [18], [19], [20], [21].

In addition, the sorting of Gap1 to the plasma membrane in the secretion pathway is also regulated by amino acid availability. Gap1-containing secretion vesicles are directed towards the vacuole-lysosome upon arrival of amino acids [22], [23]. The sophisticated, multi-layered control on Gap1 trafficking exerted by amino acid availability, suggests that amino acid import may be toxic under specific conditions [24]. Alternatively, in view of the receptor function of Gap1 [25], it may be interpreted in line with the well-established ligand-induced downregulation of eukaryotic plasma membrane receptors by internalization and sorting to the lysosome [26]. In the same way, Gap1 internalization may prevent overstimulation of the PKA pathway, a condition well known to cause loss of growth capacity and viability in yeast cells [27].

For its transport and signaling function, as well as for the control of its complex intracellular trafficking, Gap1 must interact with a large number of proteins. Many of these, for instance those involved in the Gap1 signaling function, remain to be identified. The same or similar proteins may be involved in ammonium-induced signaling by Mep2 and in its membrane targeting during nitrogen starvation. We have now made use of the split-ubiquitin based yeast two-hybrid system [28], [29] to identify proteins physically interacting with the Gap1 and/or Mep2 transceptors. This system is a modified yeast two-hybrid system, specifically designed for the identification of proteins that interact with membrane proteins and makes use of the ubiquitin protein. Ubiquitin is a conserved protein usually attached to the N-terminus of a target protein in order to mark it for degradation. Ubiquitin-tagged proteins are recognized by ubiquitin-specific proteases (UBPs), resulting in cleavage between the C-terminal residue of ubiquitin and the target protein. Ubiquitin consists of an N-terminal (Nub) and a C-terminal (Cub) domain. When Nub and Cub are expressed as separate polypeptide chains in the same cell, they will re-associate and form a quasi-native, functional ubiquitin. Replacing Ile-13 of Nub with glycine (NubG) decreases the affinity between Nub and Cub, and as a consequence, the association is now dependent on additional contacts between the fused proteins. In addition, a hybrid transcription factor is attached to the C-terminus of the Cub moiety. This transcription factor consists of the bacterial DNA-binding protein LexA, followed by the transcription activation domain of *Herpes simplex* VP16. It can activate the transcription of the *HIS3* and *LacZ* reporter genes. A fusion of the Cub moiety and the hybrid transcription factor is attached to the membrane protein of interest, Gap1 or Mep2. Next to this, a second protein fusion is used consisting of protein X and the NubG moiety. Both fusion proteins are expressed in the same strain. When protein X interacts with Gap1 or Mep2, the NubG and Cub domains are in close proximity and reconstitute a quasi-native split-ubiquitin, which is recognized by UBPs resulting in the release of the transcription factor and subsequent expression of the reporter genes. Our screen yielded a large number of candidate interacting proteins for both Gap1 and Mep2. For part of the proteins, the interaction could be confirmed by GST pull-down. Hence, the screen has provided several candidate proteins involved in the transport and signaling functions or in the complex trafficking control of these transceptors. The two proteins with unknown function, Bsc6 and Yir014w, appear to be involved in trafficking or downregulation of Gap1. On the other hand, deletion of *EGD2*, *YNL024c* or *SPC2* reduced the transport and signaling functions of Gap1, without reducing the level of the protein in the plasma membrane. The vacuolar proton-ATPase, Vma4, is required for proper expression of the *MEP2* gene, and the plasma membrane protein Gup1 seems to be required for proper homogenous distribution of Mep2 over the plasma membrane.

## Results

### Screen with cDNA library

We screened a cDNA library using a split-ubiquitin, LexA based yeast two-hybrid system for putative interacting proteins with Gap1 and/or Mep2 using the high expression plasmids pTMBV-Gap1 or pTMBV-Mep2 as bait vectors. On several occasions, independently isolated prey clones contained identical cDNA inserts. Separate retransformation of the prey vectors into the bait-containing reporter strain was used to confirm the interaction. For Gap1, we isolated 62 unique genes encoding putative interaction partners. Of these 62 genes, we could confirm the interaction for 36 genes after retransformation of the prey vector in the pTMBV-Gap1 containing reporter strain (**Table S2**). For Mep2, we isolated 36 unique genes encoding putative interaction partners. Of these 36 genes, we could confirm the interaction for 26 genes after retransformation of the prey vector in the pTMBV-Mep2 containing reporter strain (**Table S3**). Nine confirmed interaction partners (Egd2, Hyp2, Lip1, Pho88, Tpi1, Tsc13, Vma9, Vtc1 and Vtc4) were isolated using both Gap1 and Mep2 as a bait.

### Requirement for Gap1 or Mep2 transport and signaling capacity

After identification of the putative interacting proteins, we evaluated their possible requirement for Gap1- or Mep2-dependent transport and/or signaling. For this purpose, the haploid deletion strains of the systematic yeast deletion collection were used. For essential genes, the heterozygous diploid strain lacking one of the two alleles of this gene was used. As in previous work [1], [8], signaling capacity to the PKA pathway was measured using the rapid increase in activity of trehalase, a well-established PKA target [6], [7], [30], [31], after addition of 10 mM of L-citrulline or 10 mM of NH_4_^+^ respectively to nitrogen-starved fermentative cells. Similarly, transport activity was measured after addition of 10 mM of [C^14^]-labeled L-citrulline or 1 mM [C^14^]-labeled methylamine, an ammonium analogue that is transported by the Mep carriers. The signaling capacity and transport activity in the deletion mutant are always compared with those in a wild type and a *gap1Δ* or *mep1Δ mep2Δ mep3Δ* strain, respectively.

For many of the gene products identified as putatively interacting with Gap1 and/or Mep2, deletion of the corresponding gene affects transport and/or signaling capacity, i.e. reduction to less than 60% of the wild type activity. Tables 1 and 2 give an overview of all gene deletions, which cause an effect on transport and/or signaling capacity for Gap1 and Mep2, respectively. In most, but interestingly not all cases, transport and signaling are affected in a similar way. For all other putative interactors, the haploid deletion or heterozygous deletion (in case of essential genes) has no effect on Gap1- or Mep2-dependent transport and signaling.

**Table 1:** Gap1-interacting proteins of which the deletion affects Gap1-dependent transport and/or signaling activity.

		Citrulline uptake rate (% of wild type)	Trehalase activation (% of wild type)
**Both transport and signaling reduced**			
Dpm1	het dipl	8.8±2.2%	17.0±4.4%
Bsc6	hapl del	37.0±5.7%	44.0±8.7%
Egd2	hapl del	27.9±5.3%	42.0±3.5%
Ynl024C	hapl del	26.9±6.2%	46.0±10.0%
Yir014w	hapl del	49.0±1.3%	50.0±2.0%
Ted1	hapl del	17.9±3.9%	13.0±3.0%
Srp102	het dipl	36.6±8.6%	41.3±6.1%
Spc2	hapl del	55.9±9.1%	49.0±2.1%
Tpi1	het dipl	11.3±3.0%	51.5±4.9%
Lip1	het dipl	44.1±9.7%	26.0±3.5%
Pis1	het dipl	23.0±4.4%	33.6±4.0%
**Transport ± normal, but signaling reduced**			
Tsc13	het dipl	74.4±9.8%	51.7±7.6%
Sss1	het dipl	68.9±2.7%	26.7±4.2%
**Reduced transport, but signaling not affected**			
Nhx1	hapl del	18.5±5.0%	98.0±8.5%
Cwp2	hapl del	50.6±5.5%	85.0±1.4%
Pmp3	hapl del	20.1±3.4%	102.0±5.6%
**Transport and/or signaling enhanced**			
Fmp46	hapl del	203.5±9.2%	96.5±4.9%
Fks1	hapl del	177.5±5.3%	305.0±7.6%

Both trehalase activation and the L-citrulline uptake rate are expressed as percentage compared to that in the wild type strain. For transport, the average of three independent experiments was taken and standard deviation is shown. For trehalase, the average of the maximal activity (with subtraction of the mean of the blank values) after addition of 10 mM L-citrulline, was compared between the deletion strain and the wild type. (het dipl: heterozygous diploid strain, hapl del: haploid deletion strain).

**Table 2:** Mep2-interacting proteins of which the deletion affects Mep2-dependent transport and/or signaling activity.

		Methylamine uptake rate(% of wild type)	Trehalase activation(% of wild type)
**Transport increased, signaling normal**			
Phs1	het dipl	204.0±9.3%	93.0±9.8%
Zeo1	het dipl	138.0±9.2%	101.6±4.4%
Hxt2	hapl del	135.5±14.9%	93.0±1.5%
Cbf5	het dipl	235.0±2.2%	97.0±10.8%
**Reduced transport, signaling increased**			
Vma4	hapl del	17.7±1.1%	281.0±22.5%
**Transport and/or signaling increased**			
Gup1	hapl del	114.0±8.5%	206.0±11.0%
Pmt1	hapl del	121.0±6.0%	204.0±9.6%
Vtc1	hapl del	125.7±2.9%	160.0±14.0%
Vtc4	hapl del	134.9±4.1%	151.0±13.0%

Both trehalase activity and the methylamine uptake rate are expressed as percentage compared to that in the wild type strain. For transport, the average of three independent experiments was taken and standard deviation is shown. For trehalase, the maximal activity (with subtraction of the mean of the blank values) measured 10 min after addition of 10 mM NH_4_^+^, was compared between the deletion strain and the wild type. (het dipl: heterozygous diploid strain, hapl del: haploid deletion strain).

**Figure 1:**
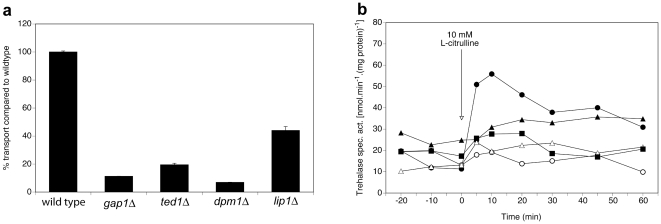
Requirement of Ted1, Dpm1 and Lip1 for Gap1-dependent transport and signaling. (**a**) Transport of 10 mM L-citrulline in nitrogen-starved cells of the *ted1Δ, dpm1Δ* and *lip1Δ* strains compared to the wild type and *gap1Δ* strains. (**b**) Activity of the PKA target trehalase after addition of 10 mM L-citrulline to nitrogen-starved cells of wild type (λ), *gap1Δ* (○), *ted1Δ* (▴), *dpm1Δ* (*Δ*) and *lip1Δ* (▪) strains.

For eleven of the putative Gap1 interactors: Dpm1, Bsc6, Egd2, Ynl024c, Yir014w, Ted1, Srp102, Spc2, Tpi1, Lip1 and Pis1, deletion of the corresponding gene results in reduction of both Gap1-dependent transport and signaling, as shown in Figure 1 for Ted1, Dpm1 and Lip1.

**Figure 2:**
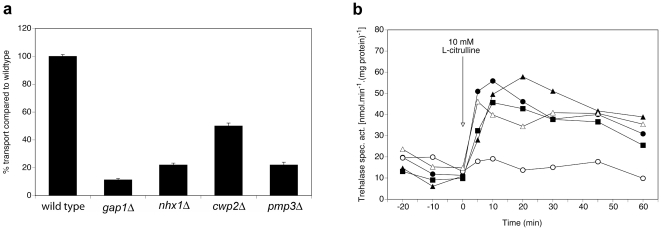
Requirement of Nhx1, Cwp2 and Pmp3 for Gap1-dependent transport and signaling. (**a**) Transport of 10 mM L-citrulline in nitrogen-starved cells of the *nhx1Δ, cwp2Δ* and *pmp3Δ* strains compared to the wild type and *gap1Δ* strains. (**b**) Activity of the PKA target trehalase after addition of 10 mM L-citrulline to nitrogen-starved cells of wild type (λ), *gap1Δ* (○), *nhx1Δ* (▴), *cwp2Δ* (Δ) and *pmp3Δ* (▪) strains.

For other interactors, deletion has no effect on Gap1-dependent signaling, i.e. trehalase activation in these mutants is similar to that in the wild type strain, however the transport rate is affected. For example, deletion of *NHX1*, *CWP2* or *PMP3* results in strongly reduced transport activity of Gap1, while signaling is not affected, as shown in Figure 2 for Nhx1, Cwp2 and Pmp3.

**Figure 3:**
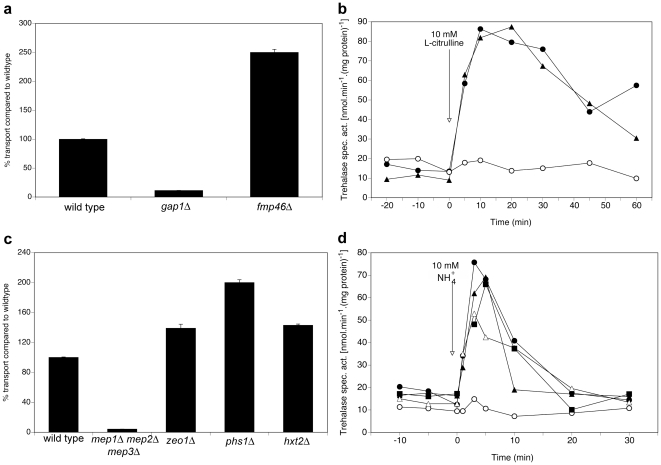
Requirement of Fmp46 for Gap1-dependent transport and signaling, and Zeo1, Phs1 and Hxt2 for Mep2-dependent transport and signaling. (**a**) Transport of 10 mM L-citrulline in nitrogen-starved cells of the *fmp46Δ* strain compared to the wild type and *gap1Δ* strains. (**b**) Activity of the PKA target trehalase after addition of 10 mM L-citrulline to nitrogen-starved cells of wild type (λ), *gap1Δ* (○) and *fmp46Δ* (▴) strains. **c**) Transport of 1 mM methylamine in nitrogen-starved cells of the *zeo1Δ, phs1Δ* and *hxt2Δ* strains compared to the wild type and *mep1Δ mep2Δ mep3Δ* strains. (**d**) Activity of the PKA target trehalase after addition of 10 mM NH_4_^+^ to nitrogen-starved cells of wild type (λ), *mep1Δ mep2Δ mep3Δ* (○), *zeo1Δ* (▴), *phs1Δ* (Δ) and *hxt2Δ* (▪) strains.

For another category of interactors, deletion increases the Gap1 transport capacity, but does not affect the signaling capacity, as was the case for the Gap1 interactor Fmp46, and the Mep2 interactors Phs1, Zeo1, Hxt2 and Cbf5, as shown in Figure 3 for Fmp46 and Zeo1, Phs1 and Hxt2.

**Figure 4:**
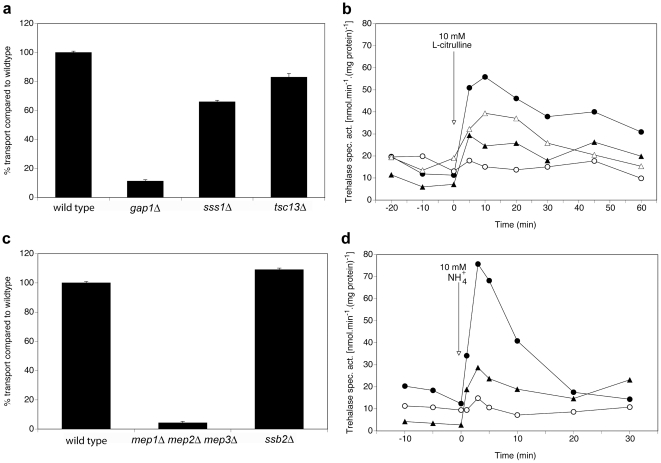
Differential requirement of Sss1 and Tsc13 for Gap1-dependent transport and signaling and Ssb2 for Mep2-dependent transport and signaling. (**a**) Transport of 10 mM L-citrulline in nitrogen-starved cells of the *sss1Δ* and *tsc13Δ* strains compared to the wild type and *gap1Δ* strains. (**b**) Activity of the PKA target trehalase after addition of 10 mM L-citrulline to nitrogen-starved cells of wild type (λ), *gap1Δ* (○), *sss1Δ* (▴) and *tsc13Δ* (Δ) strains. (**c**) Transport of 1 mM methylamine in nitrogen-starved cells of the *ssb2Δ* strain compared to the wild type and *mep1Δ mep2Δ mep3Δ* strains. (**d**) Activity of the PKA target trehalase after addition of 10 mM NH_4_^+^ to nitrogen-starved cells of wild type (λ), *mep1Δ mep2Δ mep3Δ* (○) and *ssb2Δ* (▴) strains.

In the case of the putative interactors, Tsc13, Sss1 for Gap1, and Ssb2 for Mep2, deletion results in reduced signaling, while the transport capacity remains the same, as shown in Figure 4 for Sss1, Tsc13 and Ssb2.

**Figure 5:**
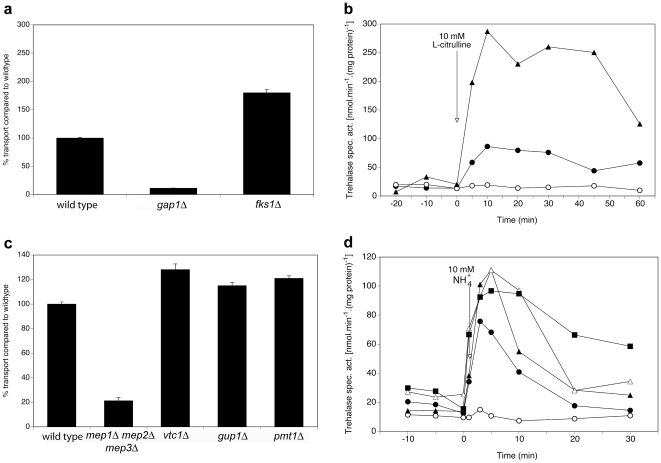
Differential requirement of Fks1 for Gap1-dependent transport and signaling, and Vtc1, Gup1 and Pmt1 for Mep2-dependent transport and signaling. (**a**) Transport of 10 mM L-citrulline in nitrogen-starved cells of the *fks1Δ* strains compared to the wild type and *gap1Δ* strains. (**b**) Activity of the PKA target trehalase after addition of 10 mM L-citrulline to nitrogen-starved cells of wild type (λ), *gap1Δ* (○) and *fks1* (▴) strains. (**c**) Transport of 1 mM methylamine in nitrogen-starved cells of the *vtc1Δ,* (▴), *gup1Δ* and *pmt1Δ* strains compared to the wild type and *mep1Δ mep2Δ mep3Δ* strains. (**d**) Activity of the PKA target trehalase after addition of 10 mM NH_4_^+^ to nitrogen-starved cells of wild type (λ), *mep1Δ mep2Δ mep3Δ* (○) and *vtc1Δ* (▴), *gup1Δ* (Δ) and *pmt1Δ* (▪) strains.

The last category of interactors causes upon deletion an increase of both transport and signaling compared to the wild type strain (Figure 5). This is the case for the Gap1 interactor Fks1 and the Mep2 interactors Gup1, Pmt1, Vtc1 and Vtc4 as shown in Figure 5 for Fks1 and Vtc1, Gup1 and Pmt1.

**Figure 6:**
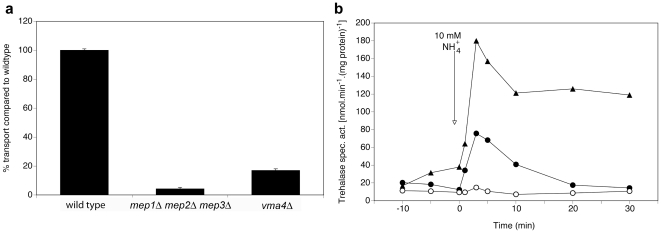
Requirement of Vma4 for Mep2-dependent transport and signaling. (**a**) Transport of 1 mM methylamine in nitrogen-starved cells of the *vma4Δ* strain compared to the wild type and *mep1Δ mep2Δ mep3Δ* strains. (**b**) Activity of the PKA target trehalase after addition of 10 mM NH_4_^+^ to nitrogen-starved cells of wild type (λ), *mep1Δ mep2Δ mep3Δ* (○) and *vma4Δ* (▴) strains.

One of the Mep2 interactors, Vma4, affects both transport and signaling but in an opposite manner. Whereas deletion of *VMA4* results in severely reduced transport rates, the signaling capacity is strongly increased (Figure 6).

### Expression and plasma membrane localization of the transceptors in deletion mutants affected in the function(s) of the transceptors

For all cases where deletion of the putative interacting proteins affects transport and/or signaling, gene expression and plasma membrane localization were analyzed by RT-PCR or by fluorescence microscopy, respectively. In contrast to the determination of transport and signaling capacity, the strains used were segregants from a cross between the haploid deletion strains of the systematic yeast deletion collection and the wild type strain with Gap1 or Mep2 tagged with mCherry in the genome. For this reason, the diploid deletion strains of essential genes were not included in the assay (*DPM1*, *SRP102*, *TPI1*, *LIP1* and *PIS1,* in the case of Gap1 and *PHS1* and *CBF5,* in the case of Mep2). The *GAP1* or *MEP2* expression levels as well as the plasma membrane localization in nitrogen-starved cells are compared with the expression level and localization in the corresponding nitrogen-starved wild type strain.

**Figure 7:**
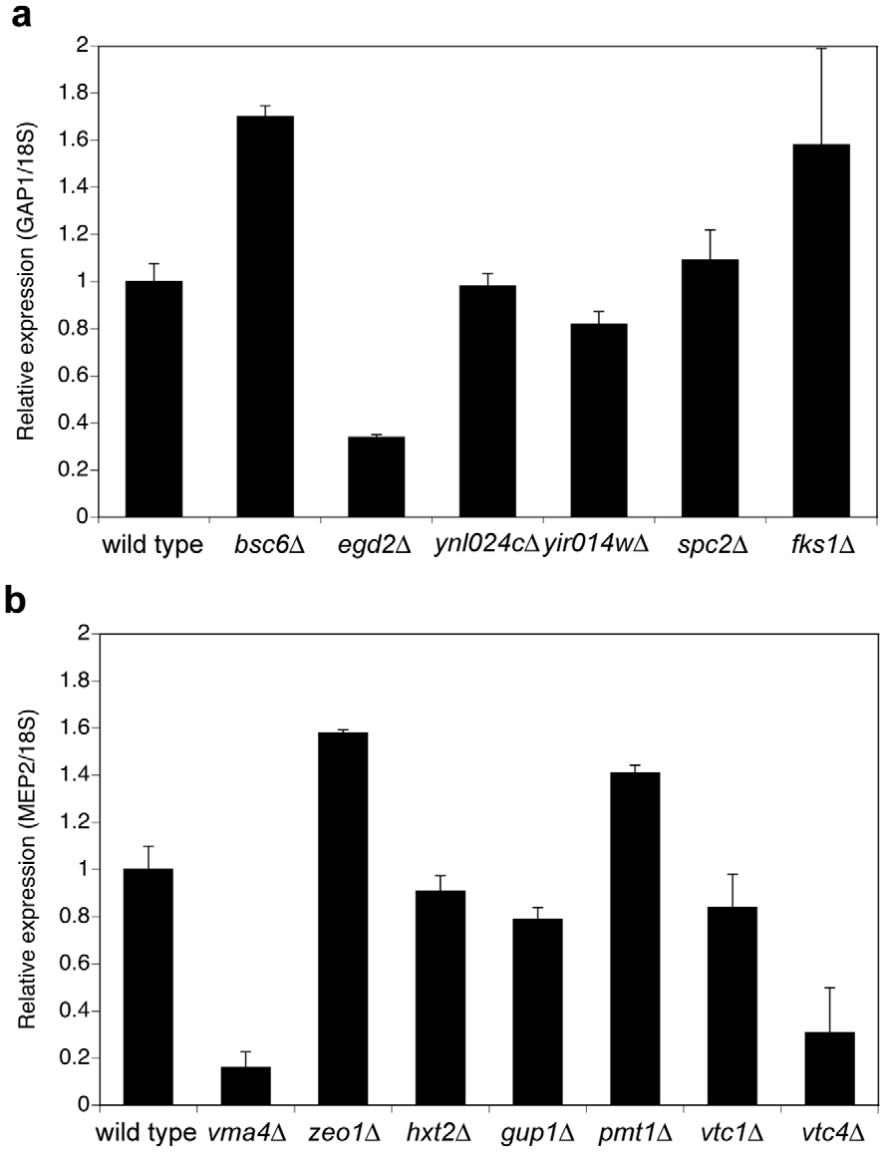
Expression of *GAP1* and *MEP2* under nitrogen starvation conditions. Relative expression levels of *GAP1* (**a**), and *MEP2* (**b**) in nitrogen-starved cells. Expression levels are normalized to the housekeeping gene *18S* and are shown relative to the levels in the wild type strain (used as the reference sample) as calculated by the comparative Ct method.

For most deletion strains, the expression of *GAP1* or *MEP2* is comparable to that in the wild type strain (Figure 7). In the case of *MEP2,* only for the *vma4Δ* and the *vtc4Δ* strains expression is strongly reduced. For the *vma4Δ* strain, this explains why only a very weak Mep2-mCherry signal was observed at the plasma membrane (results not shown), apparently causing the very low transport capacity. In the *vtc4Δ* strain, however, in spite of the reduced gene expression level, the GFP signal is comparable to that of the wild type strain. In the case of *bsc6Δ* and *fks1Δ* for *GAP1*, and *zeo1Δ* and *pmt1Δ f*or *MEP2,* expression is increased compared to the wild type strain. An increase in gene expression does not per se result in a higher protein level, as can be concluded from the protein localization studies.

**Figure 8:**
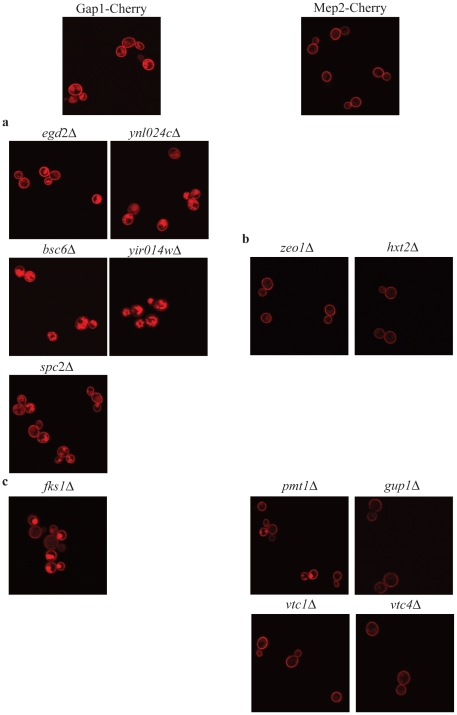
Localization of Gap1-mCherry and Mep2-mCherry, in nitrogen-starved cells of specific deletion mutants. The left column represents Gap1 localization, the right column represents Mep2 localization.

For most deletion strains, Gap1 or Mep2 is observed at the plasma membrane in levels comparable to those in the wild type strain (Figure 8). However, deletion of *BSC6* or *YIR014w*, reduces Gap1 levels at the plasma membrane and increases the presence of Gap1 in intracellular vesicles, as compared to the wild type strain (Figure 8a), in agreement with the reduction observed for both transport and signaling of Gap1.

For two of the interactors of which deletion selectively increases transport, *ZEO1* and *HXT2*, we observed plasma membrane localization of Gap1 similar to that of the wild type strain (Figure 8b). Phs1 and Cbf5 were not included because they are essential genes.

In Figure 8c, the localization of Gap1 (left) and Mep2 (right) are shown for deletion strains resulting in increased transport and signaling. For none of these strains localization of the transceptors at the plasma membrane is increased. However, in the case of *fks1*Δ and *pmt1Δ*, the vacuolar signal is strongly enhanced, suggesting a higher turnover of Gap1 and Mep2, respectively, due to higher gene expression. In the case of *gup1Δ*, Mep2 localization at the plasma membrane is aberrant. Instead of a continuous signal over the entire plasma membrane, Mep2 is localized in patches.

### Confirmation of interaction by GST pull-down assay

To confirm the protein-protein interactions identified by the split-ubiquitin screen, we performed GST pull-down assays for all interactors that have an effect on transport and/or signaling. A GST fusion of the C-terminal tail of Gap1 (Lys^548^-Cys^602^) or Mep2 (Pro^419^-Val^499^) (bait) was expressed in *E.coli*, purified and incubated with yeast extract containing an HA-tagged interacting (prey) protein. The prey constructs contained fragments of the complete ORF of the respective proteins, with the resulting protein fragments, used in the pull-down assay, ranging in size between 14 and 15 kDa. We used GST-fusion constructs of the C-terminal tail of Gap1 or Mep2, since for plasma membrane proteins, full-length proteins cannot be used for expression in *E.coli*. Since interactions might also take place at another site in the protein, a negative result does not exclude direct interaction with Gap1 or Mep2.

**Figure 9:**
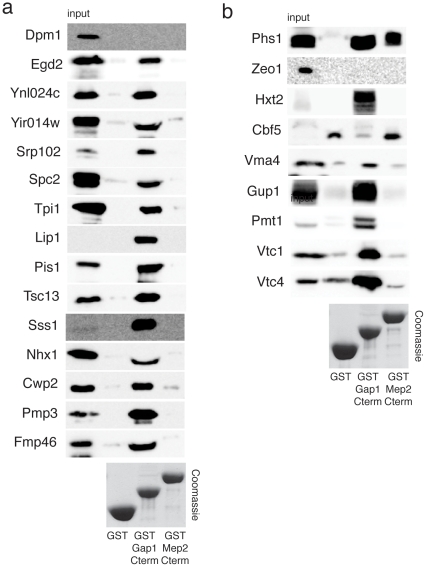
Confirmation of most interactions by GST pull-down assay using the C-terminal tail of Gap1 and Mep2. The GST fusion proteins were purified from bacteria using glutathione-Sepharose beads and incubated with cell extracts from strain NMY32 expressing the interacting cDNA clones. (**a**) A GST pull-down assay of the Gap1 interactors with the C-terminal tail of Gap1 and Mep2. (**b**) A GST pull-down assay of the Mep2 interactors with the C-terminal tail of Gap1 and Mep2.

For Gap1, the pull-down results confirm the interaction for most proteins identified in the split-ubiquitin screen that cause an effect on signaling and/or transport: Egd2, Ynl024c, Yir014w, Srp102, Spc2, Tpi1, Lip1, Pis1, Tsc13, Sss1, Nhx1, Cwp2, Pmp3 and Fmp46 (Figure 9a). Only for the dolichol-phosphate mannose synthase, Dpm1, the interaction with Gap1 could not be confirmed by the pull-down assay. For three other interactors (Bsc6, Ted1 and Fks1) we were unable to confirm the interaction reliably due to the absence of a clear input signal (results not shown).

For Mep2, the pull-down results are not straightforward (Figure 9b). Only for Phs1 and Cbf5 a clear band shows up with the Mep2 C-terminal tail. Phs1 also interacts with the Gap1 C-terminal tail, indicating that Phs1 is not a Mep2-specific interactor and may actually interact with multiple nutrient permeases. For Cbf5, a non-specific interaction was observed with the GST control. However, no interaction was observed with the GST-Gap1 C-terminal tail indicating that the interaction with Mep2 might be specific. For the other Mep2 interactors (Vma4, Hxt2, Gup1, Pmt1, Vtc1 and Vtc4), no or only weak interaction with the Mep2 C-terminal tail was observed, in spite of strong interaction with the Gap1 C-terminal tail. Vtc1 and Vtc4 were initially also isolated as Gap1 interactors, which fits with the positive result in the pull-down assay with the C-terminal tail, but deletion of the corresponding genes had no effect on transport and/or signaling. A possible explanation for the absence of interaction with the Mep2 C-terminal tail is that the interaction might occur at a different site in the protein. A potential interaction with the N-terminus is unlikely, since, in the case of Mep2, this tail is not located in the cytoplasm. Another explanation could be that interaction with the C–terminal tail of Mep2 needs specific post-translational modifications that do not take place in *E.coli*. Alternatively, the interactions might require a specific conformation of the C-terminal tail, which is lost due to its separation from the rest of the protein. For Gap1, such post-translational modifications or specific conformation might not be required for interaction. This could also explain the lack of interaction of Mep2 with Egd2, Tpi1, Lip1 and Tsc13, proteins identified as interactors of both Gap1 and Mep2 in our split-ubiquitin screen, but only found to interact with the Gap1 C-terminal tail in vitro (Figure 9a).

## Discussion

Using a split-ubiquitin yeast two-hybrid screen with Gap1 or Mep2 as bait, several candidate interacting proteins were identified. They can be subdivided into different classes according to their cellular function (**Tables S1, S2**). The overlap in cellular function between the Gap1 and Mep2 putative interactors is striking, as well as the differential effects on transport, signaling and localization of either Gap1 or Mep2.

It has to be emphasized that the split-ubiquitin screen uses cell growth as a read-out and that the Gap1 and Mep2 proteins were expressed from constitutive promoters in conditions were they are normally not or only weakly expressed. Our screen used a cDNA library constructed using mRNA from cells grown in exponential phase. This does not allow the identification of interactors, which are specifically expressed under nitrogen starvation conditions, in which the expression of *GAP1* and *MEP2* is the highest. Also proteins that require for their interaction specific post-translational modifications of Gap1 or Mep2 (e.g. phosphorylation), that are made only under the regular expression conditions of nitrogen limitation, will not be picked up in the screen.

### Interactors of which deletion has no effect on the transport and signaling function

For many of the putative interactors, deletion does not affect transport and signaling, indicating that they are either not involved in the signaling or the transport function or that they are redundant with (an)other protein(s). This was the case for all ribosomal proteins, and proteins involved in initiation or elongation of translation. These interactions probably take place during the synthesis of the transceptor proteins and thereby do not affect the functionality of the protein in the plasma membrane.

The same is true for most identified plasma membrane proteins, such as Vma9 and Pho88, interactors for both Gap1 and Mep2, and the Gap1 interactor Pho89. Interaction of these plasma membrane proteins with Gap1 or Mep2 most likely occurs during the synthesis in the ER, along the secretory pathway or at the plasma membrane, without having an effect on transceptor functionality. It was suggested that Pho88 acts as a chaperone promoting maturation or trafficking of phosphate transporters. The identification of Pho88 as an interactor of both Gap1 (also reported by [32]) and Mep2 is striking and may indicate that Pho88 functions as a chaperone for multiple membrane permeases during their trafficking through the secretory pathway. The absence of any effect of *PHO88* deletion on Gap1 and Mep2 functionality may be due to the presence of multiple chaperone proteins with a redundant function.

### Gap1 interactors required for both the transport and signaling function

Deletion of many of the Gap1 interactors strongly affects both the transport and signaling function of Gap1. Previous work has shown that any reduction of Gap1 protein levels in the plasma membrane reduces both transport and signaling [1]. This is confirmed in the case of *BSC6* and *YIR014w* deletion, where Gap1 is less present at the plasma membrane and more abundant in intracellular vesicles. These interactors might be involved in delivery of functional Gap1 to the plasma membrane. For the other interactors of this category: Egd2, Ynl024c and Spc2, deletion results in a localization pattern that is similar to wild type, but both transport and signaling of Gap1 were reduced. Hence, these proteins may in some way be required for the proper folding and/or post-translational modification of Gap1.

Although not much is known on possible glycosylation of Gap1, the identification of the dolichol-phosphate mannose synthase (Dpm1) as a putative interactor indicates that Gap1 might be modified by N-glycosylation. Since deletion of *DPM1* affects both Gap1 mediated transport and signaling, N-glycosylation might be important for the correct conformation of Gap1, and as a consequence, also for correct localization and functionality of the protein.

Similar results were obtained for interactors involved in sphingolipid biosynthesis (Pis1 and Lip1). The essential phospholipid phosphatidylinositol (PI) is synthesized by the PI synthase, Pis1 [33]. In addition to a structural role in membranes, PI is also a precursor of complex sphingolipids [34]. All sphingolipids are composed of long-chain bases (LCB), a fatty acid and a polar head group. In *S. cerevisiae*, the fatty acid is a C26-very long chain fatty acid (C26-VLCFA). Together with the long chain base phytosphingosine, C26-VLCFA forms ceramide, a reaction catalyzed by the ceramide synthase complex. Lip1 is one of the subunits of this ER-localized enzyme [35], [36]. Previous experiments indicated that addition of phytosphingosine (PHS) reduces the uptake of multiple nutrients, a.o. by reduction of the level of Gap1 in the plasma membrane [37]. Recent findings by the group of André, suggest that *de novo* synthesis of sphingolipids is required for plasma membrane stabilization of newly synthesized Gap1 [38]. Since Lip1 is involved in the synthesis of ceramides, its deletion results in accumulation of dihydrosphingosine (DHS), for which an inhibitory effect on Gap1 activity was shown [38]. In addition, it has been shown that absence of *ELO3*, which impairs formation of C26-VLCFA, results in increased endocytosis and degradation of Pma1 [39].

### Gap1 interactors of which deletion only reduces transport capacity

Two out of the three proteins of which the absence caused a reduction in Gap1 transport but did not affect signaling, are involved in maintenance of the membrane potential. These are the endosomal Na^+^/H^+^ exchanger Nhx1 and the small plasma membrane protein Pmp3. Pmp3 was previously shown to interact with permeases, such as the Agp1 amino acid permease with broad substrate specificity, the maltose permease Mal31 and the S-methylmethionine permease Mmp1 [32]. Deletion of *PMP3* is known to result in enhanced transport of methylammonium as a consequence of plasma membrane hyperpolarization [40]. Since Gap1 is an amino acid/proton symporter, the membrane potential is important for its transport activity. However, transport through Gap1 is not required for signaling [32], which may explain why deletion of *PMP3* only affects the transport function. Interestingly, the Mep2 interactor Ydr307w was found in a screen by Miller et al (2005) to interact with Pmp3 and Shr3, two proteins identified as Gap1 interactors in our screen and in previous studies [41]. This indicates that Pmp3 and its interactors might be important for regulation of the transport function of both Gap1 and Mep2, and possibly of other permeases as well.

The same differential effect is also caused by the cell wall mannoprotein, Cwp2. Deletion of *CWP2* is known to significantly reduce the thickness of the electron-dense outer layer of the cell wall and increases cell permeability [42], [43]. Therefore, deletion of this protein might indirectly alter the plasma membrane potential or composition, thereby affecting the transport but not the signaling function of Gap1. This differential effect on transport and signaling confirms our previous finding that transport is not required for signaling. Only recognition, i.e. binding of the substrate to Gap1, is required for signaling [4].

### Interactors of which deletion only increases the transport capacity

Deletion of a few interactors results in an increase in transport, without affecting the signaling function of the transceptors. This was the case for the Gap1 interactor Fmp46, and the Mep2 interactors Phs1, Zeo1, Hxt2 and Cbf5. This differential effect again confirms that both functions of the transceptors can operate independently. Since an increase in the level of Gap1 in the plasma membrane enhances both transport and signaling [1], it is unlikely that deletion of these proteins enhances the transport capacity by increasing the amount of the transceptor in the plasma membrane, as was confirmed by the localization experiment. Probably, deletion more likely increases transport activity because of altered physical interaction.

Since Zeo1 is part of the cell wall integrity (CWI) pathway, its deletion might influence the structure of the cell wall, which may in turn affect the transport capacity of the permeases, as observed for deletion of the cell wall protein Cwp2. Another explanation might be that modifications in cell integrity affect the plasma membrane potential or composition, which in turn may affect the transport capacity of Mep2. The absence of interaction in the GST pull-down assay with the C-terminus of Mep2 or Gap1, supports a possible indirect effect on signaling.

Another interactor with the same differential effect on the two functions of Mep2 is Phs1, which is known to catalyze the third step of the elongation of VLCFA, leading to the formation of C26-VLCFA [44]. Similar to Gap1, the sphingolipids in the plasma membrane may directly affect the conformation and thus also the transport activity of Mep2. The strong interaction observed between Phs1 and the C-terminal tail of both Gap1 and Mep2, might indicate a more general role of Phs1 in regulation of the transport capacity of permeases.

### Interactors of which deletion only reduces the signaling activity of the transceptors

In the case of the putative interactors Tsc13, Sss1 for Gap1, and Ssb2 for Mep2, deletion only affects the signaling function and not the transport function. Hence, these proteins are good candidates to function or to interact with components, in the signaling pathway from the transceptor to activation of PKA.

Tsc13 is, like Phs1, also involved in the formation of VLCFA [45]. Sss1 is a subunit of the Sec61 translocation complex that forms a channel for passage of secretory proteins through the ER membrane. It is not really clear how this could specifically affect the signaling and not the transport, but one possibility is that it hampers the secretion of another protein involved in the signaling by Gap1 at the plasma membrane. Ssb2 is a molecular chaperone that may be involved in the folding of proteins in the secretion pathway [46]. This may indicate that signaling by Mep2 requires more stringent folding than the transport function, alternatively it may hint at the involvement of a protein made in the secretion pathway in the signaling process.

### Interactors of which deletion increases both transport and signaling

In case of the Gap1 interactor Fks1 and the Mep2 interactors Gup1, Pmt1, Vtc1 and Vtc4, deletion results in increased levels of both transport and signaling. One possible explanation for higher levels of activity could be an enhanced level of the transceptor protein at the plasma membrane. Since Fks1 is a cell wall protein, involved in cell wall synthesis and maintenance, the altered composition of the cell wall in an *FKS1* deletion strain may enhance retention of Gap1 in the plasma membrane, for instance by impeding the Gap1 endocytic internalization process. The same applies to Pmt1, a protein-O mannosyl transferase involved in O-glycosylation, which is essential for cell wall rigidity. Mep2 is known to be N-glycosylated [47]. However, nothing is known about possible O-glycosylation of this protein. Our results indicate that Mep2 is possibly O-glycosylated and that O-glycosylation negatively affects both transport and signaling by the transceptor. This possibility was, however, excluded in the case of the *vtc1Δ* and *vtc4Δ* strains by the mCherry localization experiments. However, in the case of *FKS1* and *PMT1* deletion, the vacuolar signal is much more pronounced for both Gap1 and Mep2. This is probably due to a higher turnover of the plasma membrane transceptors Gap1 and Mep2 due to a higher expression level. The strong interaction of the Mep2-interactors Gup1, Pmt1, Vtc1 and Vtc4 with the Gap1 rather than the Mep2 C-terminal tail in the pull-down assay, might indicate a potential broad specificity of these proteins in regulation of permease trafficking.

When *GUP1* is deleted, Mep2 is localized at the plasma membrane, but concentrated in patches instead of a continuous distribution. A wide range of phenotypes of the *gup1Δ* strain could contribute to this abnormal localization pattern, including an irregular cell wall surface, an enhanced amount of chitin and β-1,3-glucans or even modified plasma membrane permeability/organization with a reduction in phospholipids and an increase in acylglycerols [48]. Additionally, detergent resistant membrane domains (DRMs) are present in a lower amount, and Pma1, associated with these micro-domains, is found in minor quantities in whole cell extract of the *gup1Δ* strain [49]. We observe the same for the Mep2 protein in the *gup1Δ* strain, indicating that Mep2 might be associated with these domains.

### Deletion of Mep2 interactor Vma4 reduces transport capacity but increases signaling

Deletion of *VMA4*, an essential subunit of the vacuolar H^+^-ATPase [50], strongly reduces the transport activity of Mep2, while signaling is increased. This differential effect indicates that similar to Gap1, transport and signaling by Mep2 can be separated and that both functions can operate independently. The reduction in transport rate is most likely a consequence of the very low expression of *MEP2,* which is supported by the very poor plasma membrane signal that was observed in the mCherry localization experiments. The increased signaling may be an indirect effect of the modified intracellular pH due to the absence of vacuolar H^+^-ATPase activity and thus vacuolar acidification. Recent research has suggested that the cytosolic pH may be involved as a second messenger in glucose signaling to the PKA pathway and that the vacuolar ATPase may play an essential role in this respect [51]. The identification of the V-ATPase subunits as interactors (Vma4 for Mep2 and Vma9 for both Gap1 and Mep2) may indicate a similar effect in nitrogen-induced activation of the PKA pathway.

### Conclusions

This work has shown that split-ubiquitin screening with plasma membrane proteins can lead to successful identification of a range of proteins that functionally interact with the bait protein. In our work, this has been demonstrated by the effect caused by deletion of many of the prey proteins on the transport and/or signaling function of Gap1 and Mep2 and/or on the localization of the transceptors in the plasma membrane. In addition, the interaction of several identified prey proteins was confirmed by *in vitro* GST pull-down assays with the C-terminal tail of the transceptors. A range of candidate proteins are now available that may be involved in regulating the transport and signaling functions or play a role in the complex trafficking control of Gap1 and Mep2. Although further work is required to identify the precise functions of the different prey proteins, the results obtained for the deletion mutants already allow to clearly separate the two functions of the transceptors. An increase or decrease in transport is not necessarily associated with an increase or decrease in signaling. In addition, the results have confirmed the previous conclusion that transport is not required for signaling and they have provided several strong hints that the functionality of the transceptors is affected not only by the composition of the plasma membrane but also by that of the cell wall. The screening with the two transceptors also resulted in common interacting proteins indicating that they are at least in part acting and/or regulated by similar mechanisms.

## Materials and Methods

### Strains and growth media

The *Saccharomyces cerevisiae* strains used for trehalase and transport measurements as well as expression and localization analysis are isogenic to wild type strain BY4741/2/3 (**Table S1**). These experiments were performed with nitrogen-starved cells, the cells were cultured at 30°C into exponential phase (OD_600 nm_ = 1.5–2) in rich YPD medium, containing 1% (w/v) yeast extract, 2% (w/v) Bactopeptone and 2% (w/v) glucose. Exponential phase cells were harvested, suspended in nitrogen starvation medium (NSM), containing 0.17 % (w/v) Difco yeast nitrogen base without amino acids and without ammonium sulfate and 4 % glucose, and incubated under shaking for 24 h at 30°C. Care was taken that the glucose level remained high (2 %) throughout the 24 h of incubation.

### Plasmid construction

For construction of the bait plasmids, *GAP1* and *MEP2* were amplified by PCR with primer pairs designed to insert *SfiI* restriction sites (5′…GGCCNNNNNGGCC…3′) in the flanking DNA sequences with the following primers: FwGap1 (TTTGGCCGAGGCGGCCCCACACCAGAAATTCCAGATTCTATAC), RvGap1 (AGAGGCCATTACGGCCAAAAATGAGTAATACTTCTTCGTACGAG), FwMep2 (AGACGGCCATTACGGCCAAAAATGTCTTACAATTTTACAGGTACG) and RvMep2 (TTTGGCCGAGGCGGCCCCTACTATATGGTCAGTGTTCTTAG). This PCR fragment was cloned as an *SfiI* restriction fragment in the yeast split-ubiquitin pTMBV vector in such a way that the bait gene is fused with the Cub-LexA-VP16 cassette. In this plasmid the very strong *TEF1* promoter drives bait expression.

For construction of the GST-fusion plasmids, the C-terminal tails of Gap1 and Mep2 were amplified with the following primer pairs: Fw GST-CT Gap1 (GCATCCCGGGTAAGATCTATAAGAGGAATTGGAAGC), Rv GST-CT Gap1 (GCATCTCGAGTTAACTCCAGAAATTCCAGATTCTATAC), Fw GST-CT Mep2 (GCATCCCGGGTCCATTTTTAAAACTAAGATTAAGTC). This PCR fragment was cloned as a *XmaI-XhoI* restriction fragment in the pGEX-4T-1 *E.coli* expression vector for expressing GST-tagged C-termini.

### Split-ubiquitin based two-hybrid assay

A commercial yeast cDNA library (Dualsystems Biotech AG, Zurich, Switzerland) fused N-terminally to NubG was transformed into the yeast reporter strain NMY32 expressing Gap1-Cub-TF or Mep2-Cub-TF as bait. *TRP^+^ LEU^+^* transformants were selected on SD Leu^−^Trp^−^His^−^ medium containing 5 mM 3-aminotriazole (3-AT). Library transformants were isolated from positive *HIS3^+^/LacZ^+^* yeast colonies and transformed into *E. coli* TOP10 cells according to standard procedures. These plasmids were selected for sequencing. The sense isolated library plasmids were retransformed into the NMY32 yeast strain expressing either the Gap1-Cub-TF or the Mep2-Cub-TF bait. For each isolated gene, two individual colonies were tested on the selective media. When the interaction was confirmed, the gene was selected for further investigation.

### Biochemical determinations

Trehalase activity after addition of amino acids or NH_4_^+^ was determined as previously described [1]. The specific trehalase activity is expressed as nmol glucose liberated min^−1^ (mg protein)^−1^. Total amount of protein in the samples was determined using the standard Lowry method. Maximal trehalase activity was calculated by subtracting the average of the three blanks from the maximal activity in the corresponding experiment.

### Transport assays

Amino acid transport in intact cells was assayed using [^14^C]-labeled L-citrulline as previously described [1]. Transport activity is expressed as nmol amino acid transported min^−1^ (mg protein)^−1^.

Methylamine transport in intact cells was assayed using [^14^C]-labeled methylamine as previously described [2]. Transport activity is expressed as nmol methylamine transported min^−1^ (mg protein)^−1^.

### Real-time quantitative PCR

For measuring *GAP1* and *MEP2* expression, nitrogen-starved cells were collected, spun down, pellets were frozen in liquid nitrogen and stored at −80°C. Total RNA was isolated by phenol extraction and treated with RNAse free DNAse (Roche). cDNA was prepared following the instructions of the Promega AMV reverse transcriptase system. Subsequently, relative quantification of *GAP1/MEP2* and *18S* was performed using real-time PCR with a Taqman assay on a StepOnePlus Real-time PCR System (Applied Biosystems), primers: Fw Gap1 (TTGGTGCCTCCTCTGTGGAT), Rv Gap1 (CCGTGAGTCTTAATGGCAATGA), Fw Mep2 (CTGGTGCAGGATGTAACCTT), Rv Mep2 (CCCACACCATGGATAGAGTA), Fw 18S (CACTTCTTAGAGGGACTATCGGTTTC) and Rv 18S (CAGAACGTCTAAGGGCATCACA).

### Fluorescence microscopy

The fluorescence localization studies of Gap1-mCherry and Mep2-mCherry were performed with nitrogen-starved cells and carried out using an Olympus FV1000 confocal laser scanning biological microscope. Images were processed with the accompanying software, FV10-ASW 2.0.

### Expression and purification of GST-tagged proteins from *E.coli*

Proteins were expressed in *E*. *coli* strain BL21. Expression was induced by addition of 0.3 mM IPTG (final concentration) and cells were harvested and washed once with ice-cold PBS buffer. Cells were then resuspended in 5 ml of lysis buffer (PBS 1x, 0.4% Triton X-100, 2 mM MgCl_2_, 1 mM EDTA pH 8.0, 2 mM DTT, 0.2 mg/ml lysozyme and protease inhibitor mix, complete EDTA free, Roche) and incubated on ice for 15 min. Lysis was completed by 3×15 s pulses of sonication. Lysates were clarified by centrifugation for 10 min at 12,000×*g* at 4°C. The resulting supernatant fraction was incubated with 400 µl of a 50:50 slurry of glutathione sepharose beads (GE Healthcare) (pre-equilibrated in wash buffer: PBS 1x, 0.1% Triton X-100, 2 mM MgCl_2_, 1 mM EDTA, 1 mM DTT) in a rollerdrum for 1 h at 4°C. Beads were collected by centrifugation at 500×*g* for 2 min at 4°C and washed 5 times with wash buffer.

### Expression and purification of HA-tagged proteins from yeast

Cultures (of retransformants with the corresponding plasmids) were grown to mid-log phase on SD-Trp-Leu, 2% glucose. Cells were harvested and washed once with ice-cold PBS buffer. Cells were resuspended in 500 µl ice-cold lysis buffer (PBS 1x, 0.1% Triton X-100, 10% glycerol, 2.5 mM MgCl_2_, 1 mM EDTA, 1 mM DTT, 10 mM NaF, 0.4 mM Na_3_VO_4_, 0.1 mM b-glycerophosphate, containing protease inhibitor mix, complete EDTA free, Roche). Glass beads were added and cells were lysed by vigorous vortexing (4×1 min, with cooling on ice in between). Lysates were clarified by centrifugation at 12,000×*g* at 4°C for 10 min. Supernatants were transferred to a new microcentrifuge tube and centrifuged for a second time at 12,000×*g*. Clarified extracts were kept on ice for further use in pull-down assays.

### GST pull-down assay

GST fusion proteins were extracted from BL21 *E. coli* cells as described. Beads were finally resuspended in 500 µl binding buffer (PBS 1x, 0.05% Triton X-100, 0.1 mM DTT). Yeast extracts were prepared as described and clarified extracts were incubated for 30 min at 4°C with 50 µl glutathione sepharose beads (GE Healthcare) to reduce aspecific binding. Beads were collected with a brief spin at 500×*g* and the resulting supernatant was incubated with equal amounts of beadbound purified GST fusion proteins, prepared as described. After a 2 h incubation at 4°C, samples were allowed to stand for 5 min on ice. The sedimented beads were washed three times with PBS-T (PBS 1x, 0.1% Triton X-100). Finally, proteins were solubilized by adding SDS sample buffer, separated by SDS-PAGE, and visualized by Coomassie staining or immunoblotting with anti-LexA antibody.

### Reproducibility of the results

The library screen was done once and all genes identified were retransformed to confirm the interaction. All other experiments were repeated at least twice. Standard deviations are shown for comparisons between independent data points (transport measurements). Representative results are shown for comparisons between collections of interdependent data points (time course measurements). The maximal extent of trehalase activation was variable between different experiments but the differences reported between controls and samples were highly reproducible.

## Supporting Information

Table S1 **Gap1-interacting proteins isolated in the split-ubiquitin screen.**
(DOC)

Table S2 ** Mep2-interacting proteins isolated in the split-ubiquitin screen.**
(DOC)

Table S3 * S. cerevisiae* **strains (all in BY background) used in this study.**
***(DOC)***
