# Supplementary material for: A Split-Ubiquitin Two-Hybrid Screen for Proteins Physically Interacting with the Yeast Amino Acid Transceptor Gap1 and Ammonium Transceptor Mep2
Source: PLoS One. 2011 Sep 2;6(9):e24275. doi: 10.1371/journal.pone.0024275 (PMC3166329; doi:10.1371/journal.pone.0024275)
Supplement: Table S1 — Gap1-interacting proteins isolated in the split-ubiquitin screen. (DOC) [file pone.0024275.s001.doc]

**Supplementary Table S1: Gap1-interacting proteins isolated in the split-ubiquitin screen.**

The second column indicates the number of times the gene was isolated.

| **Prey** | **Number** | **General Function** | **Description** |
| --- | --- | --- | --- |
| **Rpl43b** | 2 | translation (rib) | Protein component of the large (60S) ribosomal subunit [1] |
| **Rpl12b** | 1 | translation (rib) | Protein component of the large (60S) ribosomal subunit [1] |
| **Rpl19b** | 1 | translation (rib) | Protein component of the large (60S) ribosomal subunit [1] |
| **Rpl24b** | 1 | translation (rib) | Ribosomal protein L30 of the large (60S) ribosomal subunit [1] |
| **Rps12** | 1 | translation (rib) | Protein component of the small (40S) ribosomal subunit [1] |
| **Rps28a** | 3 | translation (rib) | Protein component of the small (40S) ribosomal subunit [1] |
| **Hyp2** | 6 | translation (in/el) | Translation initiation factor eIF-5A, promotes formation of the first peptide bond [2] |
| **Sui2** | 2 | translation (in/el) | Alpha subunit of the translation initiation factor eIF2, involved in the identification of the start codon [3] |
| **Tef4** | 1 | translation (in/el) | Translation elongation factor EF-1 gamma [4] |
| **Egd2** | 2 | translation (chap) | Alpha subunit of the heteromeric nascent polypeptide-associated complex (NAC) involved in protein sorting and translocation [5] |
| **Vtc4** | 2 | secretory pathway | Vacuolar membrane protein involved in vacuolar polyphosphate accumulation; regulator of vacuolar H^+^-ATPase activity and vacuolar transporter chaperones [6] |
| **Vtc1** | 3 | secretory pathway | Vacuolar transporter chaperon (VTC) involved in distributing V-ATPase and other membrane proteins [6] |
| **Sss1** | 7 | secretory pathway | Subunit of the Sec61p translocation complex (Sec61p-Sss1p-Sbh1p) that forms a channel for passage of secretory proteins through the ER membrane [7] |
| **Spc2** | 1 | secretory pathway | Subunit of signal peptidase complex (Spc1p, Spc2p, Spc3p, Sec11p), which catalyzes cleavage of N-terminal signal sequences of proteins targeted to the secretory pathway [8] |
| **Srp102** | 1 | secretory pathway | Signal recognition particle (SRP) receptor beta subunit [9] |
| **Ted1** | 1 | secretory pathway | Conserved phosphoesterase domain-containing protein that acts together with Emp24p/Erv25p in cargo exit from the ER [10] |
| **Vma9** | 1 | transport | Vacuolar H^+^-ATPase subunit e of the V-ATPase V0 subcomplex; essential for vacuolar acidification [11] |
| **Pmp3** | 1 | transport | Small plasma membrane protein related to a family of plant polypeptides that are over-expressed under high salt concentration or low temperature, deletion causes hyperpolarization of the plasma membrane potential [12] |
| **Pho89** | 1 | transport | Na^+^/Pi co-transporter, active in early growth phase [13] |
| **Pho88** | 3 | transport | Probable membrane protein, involved in phosphate transport [14] |
| **Nhx1** | 1 | transport | Endosomal Na^+^/H^+^ exchanger, required for intracellular sequestration of Na^+^ [15] |
| **Pis1** | 2 | sphingolipid biosynthesis | Phosphatidylinositol synthase, required for biosynthesis of phosphatidylinositol, which is a precursor for polyphosphoinositides, sphingo-lipids, and glycolipid anchors for some of the plasma membrane proteins [16] |
| **Lip1** | 1 | sphingolipid biosynthesis | Ceramide synthase subunit; single-span ER membrane protein associated with Lag1p and Lac1p and required for ceramide synthase activity [17] |
| **Tsc13** | 1 | sphingolipid biosynthesis | Enoyl reductase that catalyzes the last step in each cycle of very long chain fatty acid elongation, localizes to the ER [18] |
| **Dpm1** | 1 | glycosylation | Dolichol phosphate mannose (Dol-P-Man) synthase of the ER membrane, catalyzes the formation of Dol-P-Man from Dol-P and GDP-Man required for glycosyl phosphatidylinositol membrane anchoring, O mannosylation, and protein glycosylation [19] |
| **Kre1** | 1 | cell wall synthesis | Cell wall glycoprotein involved in beta-glucan assembly [20] |
| **Cwp2** | 1 | cell wall synthesis | Covalently linked cell wall mannoprotein, major constituent of the cell wall; plays a role in stabilizing the cell wall [21] |
| **Fks1** | 1 | cell wall integrity | Catalytic subunit of 1,3-beta-D-glucan synthase, functionally redundant with alternate catalytic subunit Gsc2 [22] |
| **Fmp46** | 1 | other | Putative redox protein containing a thioredoxin fold [23] |
| **Tpi1** | 1 | other | Triose phosphate isomerase, abundant glycolytic enzyme [24]; [25] |
| **Irc22** | 1 | unknown | Putative protein of unknown function; localizes to the ER [26] |
| **Bsc6** | 1 | unknown | Protein of unknown function containing 8 putative transmembrane seqments [27] |
| **Yml018c** | 1 | unknown | Putative protein of unknown function; localizes to the membrane of the vacuole |
| **Ydr056c** | 1 | unknown | Putative protein of unknown function; localizes to ER |
| **Ynl024c** | 1 | unknown | Putative protein of unknown function with seven beta-strand methyl-transferase motif localizes to the cytoplasm |
| **Yir014w** | 2 | unknown | Putative protein of unknown function; localizes to the vacuole |
