# Supplementary material for: A Split-Ubiquitin Two-Hybrid Screen for Proteins Physically Interacting with the Yeast Amino Acid Transceptor Gap1 and Ammonium Transceptor Mep2
Source: PLoS One. 2011 Sep 2;6(9):e24275. doi: 10.1371/journal.pone.0024275 (PMC3166329; doi:10.1371/journal.pone.0024275)
Supplement: Table S2 — Mep2-interacting proteins isolated in the split-ubiquitin screen. (DOC) [file pone.0024275.s002.doc]

**Supplementary Table S2: Mep2-interacting proteins isolated in the split-ubiquitin screen.**

The second column indicates the number of times the gene was isolated.

| **Prey** | **Number** | **General Function** | **Description** |
| --- | --- | --- | --- |
| **Rpl15a** | 1 | translation (rib) | Protein component of the large (60S) ribosomal subunit [1] |
| **Rpl20b** | 1 | translation (rib) | Protein component of the large (60S) ribosomal subunit [1] |
| **Rpl39** | 1 | translation (rib) | Protein component of the large (60S) ribosomal subunit [1] |
| **Rpl40a** | 1 | translation (rib) | Fusion protein that is cleaved to yield ubiquitin and & ribosomal protein of the large (60S) ribosomal subunit [1] |
| **Rps4b** | 1 | translation (rib) | Protein component of the small (40S) ribosomal subunit [1] |
| **Rps31** | 1 | translation (rib) | Fusion protein that is cleaved to yield ubiquitin and & ribosomal protein of the large (60S) ribosomal subunit [1] |
| **Hyp2** | 3 | translation (in/el) | Translation initiation factor eIF-5A, promotes formation of the first peptide bond [2] |
| **Tef1** | 1 | translation (in/el) | Translation elongation factor EF-1α that functions in the binding reaction of aminoacyl-tRNA to ribosomes [28] |
| **Ssb2** | 2 | translation (in/el) | Cytoplasmic ATPase that is a ribosome-associated molecular chaperone, functions with J-protein partner Zuo1p; may be involved in the folding of newly-synthesized polypeptide chains; member of the HSP70 family [29] |
| **Egd2** | 5 | translation (chap) | Alpha subunit of the heteromeric nascent polypeptide-associated complex (NAC) involved in protein sorting and translocation [5] |
| **Vtc1** | 2 | secretory pathway | Vacuolar transporter chaperon (VTC) involved in distributing V-ATPase and other membrane proteins [6] |
| **Vtc4** | 2 | secretory pathway | Vacuolar membrane protein involved in vacuolar polyphosphate accumulation; regulator of vacuolar H^+^-ATPase activity and vacuolar transporter chaperones [6] |
| **Vma4** | 2 | transport | Subunit E of the eight-subunit V1 peripheral membrane domain of the vacuolar H^+^-ATPase (V-ATPase), an electrogenic proton pump found throughout the endomembrane system [30] |
| **Vma9** | 2 | transport | Vacuolar H^+^-ATPase subunit e of the V-ATPase V0 subcomplex; essential for vacuolar acidification [11] |
| **Pho88** | 1 | transport | Probable membrane protein, involved in phosphate transport [14] |
| **Gup1** | 1 | transport | Plasma membrane protein involved in remodeling GPI anchors by addition of a VLCFA; proposed to be involved in glycerol transport [31] |
| **Hxt2** | 1 | transport | High-affinity glucose transporter of the major facilitator superfamily [32] |
| **Lip1** | 1 | sphingolipid biosynthesis | Ceramide synthase subunit; single-span ER membrane protein associated with Lag1p and Lac1p and required for ceramide synthase activity [17] |
| **Tsc13** | 1 | sphingolipid biosynthesis | Enoyl reductase that catalyzes the last step in each cycle of very long chain fatty acid elongation, localizes to the ER [18] |
| **Phs1** | 1 | sphingolipid biosynthesis | Essential 3-hydroxyacyl-CoA dehydratase of the ER membrane, involved in elongation of very long-chain fatty acids; involved in sphingolipid biosynthesis and protein trafficking [33] |
| **Pmt1** | 1 | glycosylation | Protein O-mannosyltransferase, transfers mannose residues from dolichyl phosphate-D-mannose to protein serine/threonine residues [34] |
| **Vrg4** | 1 | glycosylation | Golgi GDP-mannose transporter; regulates Golgi function and glycosylation in Golgi [35] |
| **Zeo1** | 6 | cell wall integrity | Peripheral membrane protein that negatively regulates the cell integrity pathway mediated by Pkc1 and Stl2 [36] |
| **Cbf5** | 1 | other | Pseudouridine synthase catalytic subunit of box H/ACA small nucleolar ribonucleoprotein particles (snoRNPs), acts on both large and small rRNAs and on snRNA U2 [37] |
| **Tpi1** | 2 | other | Triose phosphate isomerase, abundant glycolytic enzyme [24]; [25] |
| **Ydr210w** | 1 | unknown | Putative protein of unknown function, localizes to the cell periphery |
| **Ydr307w** | 1 | unknown | Putative protein of unknown function |
