# Supplementary material for: A Split-Ubiquitin Two-Hybrid Screen for Proteins Physically Interacting with the Yeast Amino Acid Transceptor Gap1 and Ammonium Transceptor Mep2
Source: PLoS One. 2011 Sep 2;6(9):e24275. doi: 10.1371/journal.pone.0024275 (PMC3166329; doi:10.1371/journal.pone.0024275)
Supplement: Table S3 — S. cerevisiae strains (all in BY background) used in this study. (DOC) [file pone.0024275.s003.doc]

**Supplementary Table S3: *S.cerevisiae* strains (all in BY background) used in this study**

| **Strain** | **Genotype** | **Isogenic to** | **Reference** |
| --- | --- | --- | --- |
| NMY32 | MATa his3delta200 trp1-901 leu2-3, 112 LYS2::(lexAop)_4_-HIS3 URA3::(lexAop)_8_-lacZ (lexAop)_8_-ADE2 GAL4 |  | Dualsystems |
| BY4742 | *Matα his3 leu2 lys2 ura3* | S288C | [38] |
| BY4743 | *BY4741/BY4742* | S288C | [38] |
| Record nr. 16906 | *BY4742 rpl43b::KANMX* | S288C | [39] |
| Record nr. 14254 | *BY4742 rpl12b::KANMX* | S288C | [39] |
| Record nr.13053 | *BY4742 rpl19b::KANMX* | S288C | [39] |
| Record nr. 14778 | *BY4742 rpl24b::KANMX* | S288C | [39] |
| Record nr. 11666 | *BY4742 rps12::KANMX* | S288C | [39] |
| Record nr.12423 | *BY4742 rps28a::KANMX* | S288C | [39] |
| Record nr. 14930 | *BY4742 tef4::KANMX* | S288C | [39] |
| Record nr. 16422 | *BY4742 egd2::KANMX* | S288C | [39] |
| Record nr. 16780 | *BY4742 vtc4::KANMX* | S288C | [39] |
| Record nr. 10212 | *BY4742 vtc1::KANMX* | S288C | [39] |
| Record nr. 13413 | *BY4742 vma9::KANMX* | S288C | [39] |
| Record nr. 13635 | *BY4742 pmp3::KANMX* | S288C | [39] |
| Record nr. 15744 | *BY4742 pho89::KANMX* | S288C | [39] |
| Record nr. 13245 | *BY4742 pho88::KANMX* | S288C | [39] |
| Record nr. 14290 | *BY4742 nhx1::KANMX* | S288C | [39] |
| Record nr. 10516 | *BY4742 spc2::KANMX* | S288C | [39] |
| Record nr. 11432 | *BY4742 ted1::KANMX* | S288C | [39] |
| Record nr. 11122 | *BY4742 kre1::KANMX* | S288C | [39] |
| Record nr. 17026 | *BY4742 cwp2::KANMX* | S288C | [39] |
| Record nr. 15251 | *BY4742 fks1::KANMX* | S288C | [39] |
| Record nr. 15120 | *BY4742 fmp46::KANMX* | S288C | [39] |
| Record nr. 10421 | *BY4742 irc22::KANMX* | S288C | [39] |
| Record nr. 16287 | *BY4742 bsc6::KANMX* | S288C | [39] |
| Record nr. 10555 | *BY4742 yml018c::KANMX* | S288C | [39] |
| Record nr. 13992 | *BY4742 ydr056c::KANMX* | S288C | [39] |
| Record nr. 15350 | *BY4742 ynl024c::KANMX* | S288C | [39] |
| Record nr. 12350 | *BY4742 yir014w::KANMX* | S288C | [39] |
| Record nr. 21584 | *BY4742 rpl15a::KANMX* | S288C | [39] |
| Record nr. 11609 | *BY4742 rpl20b::KANMX* | S288C | [39] |
| Record nr. 17018 | *BY4742 rpl39::KANMX* | S288C | [39] |
| Record nr.1609 | *BY4742 rpl40a::KANMX* | S288C | [39] |
| Record nr. 2897 | *BY4742 rps4b::KANMX* | S288C | [39] |
| Record nr. 24116 | *BY4742 rps31::KANMX* | S288C | [39] |
| PV13 | *BY4742 ssb2::KANMX* | S288C | This study |
| Record nr. 11629 | *BY4742 vma4::KANMX* | S288C | [39] |
| Record nr. 4451 | *BY4742 gup1::KANMX* | S288C | [39] |
| Record nr. 586 | *BY4742 hxt2::KANMX* | S288C | [39] |
| Record nr. 27096 | *BY4742 phs1::KANMX* | S288C | [39] |
| Record nr. 3792 | *BY4742 pmt1::KANMX* | S288C | [39] |
| Record nr. 24591 | *BY4742 vrg4::KANMX* | S288C | [39] |
| Record nr. 6259 | *BY4742 zeo1::KANMX* | S288C | [39] |
| Record nr. 13569 | *BY4742 ydr210w::KANMX* | S288C | [39] |
| Record nr. 3666 | *BY4742 yydr307w::KANMX* | S288C | [39] |
| Record nr. 26389 | *BY4743 HYP2/hyp2::KANMX* | S288C | [39] |
| Record nr. 26804 | *BY4743 SUI2/sui2::KANMX* | S288C | [39] |
| Record nr. 20421 | *BY4743 SSS1/sss1::KANMX* | S288C | [39] |
| Record nr. 24124 | *BY4743 CBF5/cbf5::KANMX* | S288C | [39] |
| Record nr. 23257 | *BY4743 TEF2/tef2::KANMX* | S288C | [39] |
| Record nr. 25002 | *BY4743 SRP102/srp102::KANMX* | S288C | [39] |
| Record nr. 25529 | *BY4743 PIS1/pis1::KANMX* | S288C | [39] |
| Record nr. 23711 | *BY4743 TSC13/tsc13::KANMX* | S288C | [39] |
| Record nr. 20886 | *BY4743 LIP1/lip1::KANMX* | S288C | [39] |
| Record nr. 25598 | *BY4743 DPM1/dpm1::KANMX* | S288C | [39] |
| Record nr. 26690 | *BY4743 TPI1/tpi1::KANMX* | S288C | [39] |
| MRT76 | *BY4741 GAP1-mCherry HIS3* | S288C | Unpublished data |
| gV270 | *BY4741 MEP2-mCherry HIS3* | S288C | This study |
| gV234 | MATa *egd2::KanMx GAP1-mCherry leu2 lys2 met15 ura3* | S288C | This study |
| gV246 | MATα *ynl024c::KanMx GAP1-mCherry leu2 lys2 ura3* | S288C | This study |
| gV236 | MATα *spc2::KanMx GAP1-mCherry leu2 ura3* | S288C | This study |
| gV244 | MATα *bsc6::KanMx GAP1-mCherry leu2 met15 ura3* | S288C | This study |
| gV256 | MATα *vma4::KanMx MEP2-mCherry leu2 lys2 mep15 ura3* | S288C | This study |
| gV248 | MATα *yir014w::KanMx GAP1-mCherry leu2 lys2 ura3* | S288C | This study |
| gV258 | MATa *zeo1::KanMx MEP2-mCherry leu2 lys2 ura3* | S288C | This study |
| gV260 | MATα *hxt2::KanMx MEP2-mCherry leu2 ura3* | S288C | This study |
| gV242 | MATα *fks1::KanMx GAP1-mCherry leu2 lys2 ura3* | S288C | This study |
| gV262 | MATa *gup1::KanMx MEP2-mCherry leu2 lys2 ura3* | S288C | This study |
| gV264 | MATa *pmt1::KanMx MEP2-mCherry leu2 met15 ura3* | S288C | This study |
| gV266 | MATα *vtc1::KanMx GAP1-mCherry leu2 met15 ura3* | S288C | This study |
| gV268 | MATα *vtc4::KanMx GAP1-mCherry leu2 met15 ura3* | S288C | This study |

**Supplementary references**

1. Planta RJ, Mager WH (1998) The list of cytoplasmic ribosomal proteins of Saccharomyces cerevisiae. Yeast 14: 471-477.

2. Schnier J, Schwelberger HG, Smit-McBride Z, Kang HA, Hershey JW (1991) Translation initiation factor 5A and its hypusine modification are essential for cell viability in the yeast Saccharomyces cerevisiae. Mol Cell Biol 11: 3105-3114.

3. Cigan AM, Pabich EK, Feng L, Donahue TF (1989) Yeast translation initiation suppressor sui2 encodes the alpha subunit of eukaryotic initiation factor 2 and shares sequence identity with the human alpha subunit. Proc Natl Acad Sci U S A 86: 2784-2788.

4. Kinzy TG, Ripmaster TL, Woolford JL, Jr. (1994) Multiple genes encode the translation elongation factor EF-1 gamma in Saccharomyces cerevisiae. Nucleic Acids Res 22: 2703-2707.

5. Shi X, Parthun MR, Jaehning JA (1995) The yeast EGD2 gene encodes a homologue of the alpha NAC subunit of the human nascent-polypeptide-associated complex. Gene 165: 199-202.

6. Cohen A, Perzov N, Nelson H, Nelson N (1999) A novel family of yeast chaperons involved in the distribution of V-ATPase and other membrane proteins. J Biol Chem 274: 26885-26893.

7. Esnault Y, Blondel MO, Deshaies RJ, Scheckman R, Kepes F (1993) The yeast SSS1 gene is essential for secretory protein translocation and encodes a conserved protein of the endoplasmic reticulum. Embo J 12: 4083-4093.

8. Mullins C, Meyer HA, Hartmann E, Green N, Fang H (1996) Structurally related Spc1p and Spc2p of yeast signal peptidase complex are functionally distinct. J Biol Chem 271: 29094-29099.

9. Ogg SC, Barz WP, Walter P (1998) A functional GTPase domain, but not its transmembrane domain, is required for function of the SRP receptor beta-subunit. J Cell Biol 142: 341-354.

10. Haass FA, Jonikas M, Walter P, Weissman JS, Jan YN, et al. (2007) Identification of yeast proteins necessary for cell-surface function of a potassium channel. Proc Natl Acad Sci U S A 104: 18079-18084.

11. Compton MA, Graham LA, Stevens TH (2006) Vma9p (subunit e) is an integral membrane V0 subunit of the yeast V-ATPase. J Biol Chem 281: 15312-15319.

12. Navarre C, Goffeau A (2000) Membrane hyperpolarization and salt sensitivity induced by deletion of PMP3, a highly conserved small protein of yeast plasma membrane. Embo J 19: 2515-2524.

13. Martinez P, Persson BL (1998) Identification, cloning and characterization of a derepressible Na+-coupled phosphate transporter in Saccharomyces cerevisiae. Mol Gen Genet 258: 628-638.

14. Yompakdee C, Ogawa N, Harashima S, Oshima Y (1996) A putative membrane protein, Pho88p, involved in inorganic phosphate transport in Saccharomyces cerevisiae. Mol Gen Genet 251: 580-590.

15. Nass R, Cunningham KW, Rao R (1997) Intracellular sequestration of sodium by a novel Na+/H+ exchanger in yeast is enhanced by mutations in the plasma membrane H+-ATPase. Insights into mechanisms of sodium tolerance. J Biol Chem 272: 26145-26152.

16. Nikawa J, Kodaki T, Yamashita S (1987) Primary structure and disruption of the phosphatidylinositol synthase gene of Saccharomyces cerevisiae. J Biol Chem 262: 4876-4881.

17. Vallee B, Riezman H (2005) Lip1p: a novel subunit of acyl-CoA ceramide synthase. Embo J 24: 730-741.

18. Kohlwein SD, Eder S, Oh CS, Martin CE, Gable K, et al. (2001) Tsc13p is required for fatty acid elongation and localizes to a novel structure at the nuclear-vacuolar interface in Saccharomyces cerevisiae. Mol Cell Biol 21: 109-125.

19. Orlean P (1990) Dolichol phosphate mannose synthase is required in vivo for glycosyl phosphatidylinositol membrane anchoring, O mannosylation, and N glycosylation of protein in Saccharomyces cerevisiae. Mol Cell Biol 10: 5796-5805.

20. Boone C, Sommer SS, Hensel A, Bussey H (1990) Yeast KRE genes provide evidence for a pathway of cell wall beta-glucan assembly. J Cell Biol 110: 1833-1843.

21. van der Vaart JM, Caro LH, Chapman JW, Klis FM, Verrips CT (1995) Identification of three mannoproteins in the cell wall of Saccharomyces cerevisiae. J Bacteriol 177: 3104-3110.

22. Douglas CM, Foor F, Marrinan JA, Morin N, Nielsen JB, et al. (1994) The Saccharomyces cerevisiae FKS1 (ETG1) gene encodes an integral membrane protein which is a subunit of 1,3-beta-D-glucan synthase. Proc Natl Acad Sci U S A 91: 12907-12911.

23. Jung JW, Yee A, Wu B, Arrowsmith CH, Lee W (2005) Solution structure of YKR049C, a putative redox protein from Saccharomyces cerevisiae. J Biochem Mol Biol 38: 550-554.

24. Alber T, Kawasaki G (1982) Nucleotide sequence of the triose phosphate isomerase gene of Saccharomyces cerevisiae. J Mol Appl Genet 1: 419-434.

25. Compagno C, Boschi F, Ranzi BM (1996) Glycerol production in a triose phosphate isomerase deficient mutant of Saccharomyces cerevisiae. Biotechnol Prog 12: 591-595.

26. Alvaro D, Lisby M, Rothstein R (2007) Genome-wide analysis of Rad52 foci reveals diverse mechanisms impacting recombination. PLoS Genet 3: e228.

27. Namy O, Duchateau-Nguyen G, Hatin I, Hermann-Le Denmat S, Termier M, et al. (2003) Identification of stop codon readthrough genes in Saccharomyces cerevisiae. Nucleic Acids Res 31: 2289-2296.

28. Schirmaier F, Philippsen P (1984) Identification of two genes coding for the translation elongation factor EF-1 alpha of S. cerevisiae. Embo J 3: 3311-3315.

29. Pfund C, Lopez-Hoyo N, Ziegelhoffer T, Schilke BA, Lopez-Buesa P, et al. (1998) The molecular chaperone Ssb from Saccharomyces cerevisiae is a component of the ribosome-nascent chain complex. Embo J 17: 3981-3989.

30. Foury F (1990) The 31-kDa polypeptide is an essential subunit of the vacuolar ATPase in Saccharomyces cerevisiae. J Biol Chem 265: 18554-18560.

31. Holst B, Lunde C, Lages F, Oliveira R, Lucas C, et al. (2000) GUP1 and its close homologue GUP2, encoding multimembrane-spanning proteins involved in active glycerol uptake in Saccharomyces cerevisiae. Mol Microbiol 37: 108-124.

32. Ozcan S, Dover J, Johnston M (1998) Glucose sensing and signaling by two glucose receptors in the yeast Saccharomyces cerevisiae. Embo J 17: 2566-2573.

33. Schuldiner M, Collins SR, Thompson NJ, Denic V, Bhamidipati A, et al. (2005) Exploration of the function and organization of the yeast early secretory pathway through an epistatic miniarray profile. Cell 123: 507-519.

34. Strahl-Bolsinger S, Immervoll T, Deutzmann R, Tanner W (1993) PMT1, the gene for a key enzyme of protein O-glycosylation in Saccharomyces cerevisiae. Proc Natl Acad Sci U S A 90: 8164-8168.

35. Dean N, Zhang YB, Poster JB (1997) The VRG4 gene is required for GDP-mannose transport into the lumen of the Golgi in the yeast, Saccharomyces cerevisiae. J Biol Chem 272: 31908-31914.

36. Green R, Lesage G, Sdicu AM, Menard P, Bussey H (2003) A synthetic analysis of the Saccharomyces cerevisiae stress sensor Mid2p, and identification of a Mid2p-interacting protein, Zeo1p, that modulates the PKC1-MPK1 cell integrity pathway. Microbiology 149: 2487-2499.

37. Lafontaine DL, Bousquet-Antonelli C, Henry Y, Caizergues-Ferrer M, Tollervey D (1998) The box H + ACA snoRNAs carry Cbf5p, the putative rRNA pseudouridine synthase. Genes Dev 12: 527-537.

38. Brachmann CB, Davies A, Cost GJ, Caputo E, Li J, et al. (1998) Designer deletion strains derived from Saccharomyces cerevisiae S288C: a useful set of strains and plasmids for PCR-mediated gene disruption and other applications. Yeast 14: 115-132.

39. Giaever G, Chu AM, Ni L, Connelly C, Riles L, et al. (2002) Functional profiling of the Saccharomyces cerevisiae genome. Nature 418: 387-391.
